# Effect of continuous spinal anesthesia on the hemodynamics of labor analgesia in hypertensive pregnant women: a comparative, randomized clinical trial

**DOI:** 10.1186/s12871-023-02174-1

**Published:** 2023-06-13

**Authors:** Bin Han, Mingjun Xu

**Affiliations:** Department of Anesthesiology, Beijing Obstetrics and Gynecology Hospital Capital Medical University, Beijing, 100026 China

**Keywords:** LiDCO rapid, Hypertensive Disorder Complicating Pregnancy, Labor analgesia, Hemodynamics

## Abstract

**Background:**

To observe the changes in hemodynamic, stress and inflammatory responses during labor and their labor outcomes after continuous spinal anesthesia labor analgesia for hypertensive pregnant women, and to evaluate whether the continuous spinal anesthesia had any advantages compared to continuous epidural analgesia for hypertensive pregnant women and their newborns.

**Methods:**

A total of 160 hypertensive pregnant women were selected and randomly divided into continuous spinal anesthesia analgesia group (CSA group) and continuous epidural analgesia group (EA group). Participant age, height, weight and gestational week were recorded; MAP, VAS score, CO and SVR were recorded after the onset of regular uterine contractions (T_0_), 10 min after analgesia (T_1_), 30 min (T_2_), 60 min (T_3_), when the uterine opening was complete (T_4_) and when the fetus was delivered (T_5_); the duration of the first stage of labor and the second stage of labor were recorded; the number of cases of treatment with oxytocin and antihypertensive therapy, mode of delivery, eclampsia and postpartum hemorrhage were counted; pregnant women Bromage scores were recorded at T_2_. We also recorded neonatal weight, Apgar scores at 1, 5 and 10 min after birth; arterial blood gas analysis of the umbilical cord was performed in newborns; finally, TNF-α, IL-6, and cortisol in pregnant women venous blood were measured at T_0_, T_5_, and 24 h after delivery (T_7_). The number of successful compressions and the total drug dosage administered by the analgesic pump were recorded for both groups.

**Results:**

The first stage of labor in CSA was longer than EA (*P* < 0.05); the MAP, VAS and SVR value in CSA were lower than EA group at T_1_, T_3_ and T_4_ (*P* < 0.05); in contrast, the CO in CSA at T3 and T4 was higher than in EA (*P* < 0.05). The oxytocin was more often used whereas the antihypertensive drugs were less used in CSA as compared to EA. The level of TNF-α, IL-6, Cor in the CSA at T5 was lower than the EA group (*P* < 0.05), and the level of TNF-α in the CSA group at T7 was lower than the EA group (*P* < 0.05).

**Conclusion:**

For pregnant women with hypertension during pregnancy, continuous spinal anesthesia labor analgesia has no significant effect on the final mode of delivery, but shows precise analgesic effect and stabilizes circulatory system, it is recommended to perform continuous spinal anesthesia early in labor for hypertensive pregnant women, which can effectively reduce the stress reaction.

**Trial registration:**

ChiCTR-INR-17012659.

Date of registration: 13/09/2017.

## Introduction

Hypertension in pregnancy is a unique disease during pregnancy [1]. The basic pathological change is the spasm of small arteries throughout the body, which is one of the most serious complications during pregnancy [2, 3]. It is estimated that nearly 10% of pregnancies suffer from hypertension worldwide. The main symptoms are hypertension and proteinuria. Currently, the general clinical treatments include spasmolysis, decreasing blood pressure, auxiliary symptomatic as well as supportive treatment, and in some cases termination of pregnancy. The stress response caused by pain during childbirth can aggravate the pathological condition of the parturient and even cause serious complications such as eclampsia [4]. To reduce the risk of delivery, many obstetricians recommend cesarean section for hypertensive parturients to end their pregnancy in order to reduce the risk of maternal delivery, but this does not significantly benefit parturients and newborns, more importantly [5], elective cesarean section can induce severe neonatal complications such as respiratory distress syndrome and neonatal pneumonia [6].

Currently, the commonly used method of labor analgesia is continuous epidural analgesia, which requires a large amount of anesthetics [7, 8]. Continuous spinal anesthesia allows continuous injection of drugs into the subarachnoid space and acts directly on the spinal cord. Compared with traditional spinal anesthesia, it has less effect on the circulatory system, lower drug usage and more precise analgesic effect. Therefore, we think it might bring multiple advantages for hypertensive pregnant women. According to our experience, we didn’t observe any difference in the failure rate between spinal catheters and epidural catheters insertion.

In our previous study, we applied continuous spinal anesthesia and continuous epidural anesthesia for analgesia in normal laboring mothers and achieved satisfactory clinical results, and we also considered the results of Tao et al.’s study regarding the dose application of continuous spinal anesthesia [9–11].

It is known that maternal position changes, effects of anesthesia, delivery of the fetus, and delivery forceps application can cause significant fluctuations in hemodynamics, whereas obstetricians usually only monitor heart rate, blood pressure, pulse as well as blood oxygen saturation [8, 12]. The LiDCO- Rapid system is a non-invasive hemodynamic monitor that can provide various hemodynamic indexes and can be used in either mechanical or non-mechanical ventilation. In this study, the main objective was to compare the effects of continuous spinal anesthesia and continuous epidural anesthesia on labor analgesia which applied to hypertensive pregnant women. The primary objective was to compare the hemodynamic changes after labor analgesia and the secondary objective was to compare the changes in maternal stress and inflammatory response when applying either continuous spinal anesthesia or continuous epidural anesthesia for hypertensive pregnant women.

## Materials and methods

### Patients selection

This study was approved by the ethical committee of Beijing Obstetrics and Gynecology Hospitaland was registered at clinical trials registry on 13/09/2017 with registration ID: ChiCTR-INR-17012659.The informed consent was signed by every parturient after explanation of aim, risks and benefits of the study.

A total of 160 parturient, aged 22–35 years old, body weight between 55 to 85 kg, who delivered in Beijing Obstetrics and Gynecology Hospital from September 2017 to September 2020 were enrolled. They were randomly divided into CSA group and EA group with 80 cases in each group. The sample size was calculated using PASS 15.0 software, and a two-sided test with a test level of 0.05 and a test efficacy of β = 0.2 was used, and the rate of loss was calculated at 10%.

#### Inclusive criteria

①All participants were diagnosed Hypertensive Disorder Complicating Pregnancy according to the diagnostic criteria;②All parturients were classified to grade II or III according to American Society of Anesthesiologists standard;③New York Heart Association Cardiac Function Grade I;④According to the diagnosis of obstetricians, vaginal trial labor is feasible and labor analgesia is voluntary;⑤No history of cesarean section;⑥No contraindication of intraspinal anesthesia, platelet > 100 × 10^9^/L, and blood coagulation function was normal.

#### Exclusion criteria

①Parturients previous diagnosed with circulatory disorders, blood system diseases, coagulation disorders, immune system diseases and mental system diseases; ②The platelet count was less than 100 × 10^9^/L, or parturients with contraindication of intraspinal anesthesia;③Parturients with nervous system diseases;④Parturients with systemic infectious diseases. The parturients who were transferred to cesarean section were excluded.

### Diagnostic criteria for gestational hypertension and severe preeclampsia

Gestational hypertension is defined as the first occurrence of hypertension during pregnancy, systolic blood pressure ≥ 140 mmHg and (or) diastolic blood pressure ≥ 90 mmHg, urine protein is negative, and it returns to normal 12 weeks after delivery.

Severe preeclampsia is defined as: (1) continuous increase in blood pressure: systolic blood pressure ≥ 160 mmHg and/or diastolic blood pressure ≥ 110 mmHg; (2) persistent headache, visual impairment or other central nervous system abnormalities; (3) persistent upper abdominal pain and liver subcapsular hematoma or liver rupture; (4)abnormal liver enzymes: elevated blood alanine aminotransferase (ALT) or aspartate aminotransferase (AST) levels; (5)renal function impairment: urine protein > 2.0 g/24 h; oliguria (24-h urine volume < 400 ml, or hourly urine volume < 17 ml), or blood creatinine > 106 μmol/L; (6)hypoproteinemia with ascites, pleural effusion or pericardial effusion; (7)platelet count is continuously decreased and less than 100 × 10^9^/L; microvascular hemolysis indicating anemia, jaundice, or elevated blood lactate dehydrogenase (LDH) level; (8)heart failure; (9)pulmonary edema; (10) fetal growth restriction or oligohydramnios, intrauterine death, placental abruption, etc.

### Anesthesia method and data collection

All parturients undergo labor analgesia after regular uterine contractions, and continue to apply analgesic pump until placental delivery (regular uterine contractions are defined as contractions every 2–3 min, and contractions last for more than 30 s). Ringer’s solution was infused before labor analgesia, and all parturients underwent fetal heart rate monitoring and LiDCO-rapid system monitoring.

For the CSA group, parturients were placed in the left decubitus position. Continuous spinal anesthesia was given between L3 and 4, and the Pajunk continuous spinal anesthesia kit (Germany) was used to puncture the 21 G spinal anesthesia needle into the subarachnoid space, and a 3 cm catheter (25G) was placed on the head side. The first dose of ropivacaine 0.5 mg/ml + sufentanil 0.5 ug/ml within 5 ml in total, 15 min later, a continuous spinal analgesia pump was connected. The formula of the analgesic pump is 100 ml of ropivacaine 0.2 mg/ml + sufentanil 0.2 ug/ml, the background dose is 3 ml/h, the patient-controlled analgesia is 3 ml/time, and the lock time is 15 min.

The parturients in the EA group were given continuous epidural anesthesia at the interval L3 to 4, and a 3 cm catheter was placed on the head side. The dose was ropivacaine 1 mg/ml + sufentanil 0.5ug/ml for a total of 5 ml, 5 min later, the same treatment was given for another time. The analgesic pump was connected after 15 min. The analgesic pump formula was 100 ml of ropivacaine 1 mg/ml + sufentanil 0.5ug/ml, a background dose of 5 ml/h, patient-controlled analgesia 5 ml/time, and a lock time of 15 min. The parturients were instructed to press the analgesic pump when VAS ≥ 4 to maintain VAS between 2 and 4 during labor. In both groups, the infusion was stopped after delivery of the placenta and the catheter was removed before the women returned to the ward.

The LiDCO-rapid monitor was used throughout the labor process. The MAP, VAS score, CO and SVR were recorded at the time of regular uterine contraction(T_0_), 10 min after labor analgesia(T_1_), 30 min after labor analgesia(T_2_), 60 min after labor analgesia(T_3_), the full opening of uterine orifice(T_4_), fetal delivery(T_5_) and before leaving the delivery room(T_6_); The time of the first stage of labor (from the regular contraction of the uterus to the full opening of the uterine orifice) and the second stage of labor (from the full opening of uterine orifice to the delivery of the fetus) were recorded; the number of cases of oxytocin and antihypertensive therapy, mode of delivery (natural delivery, forceps midwifery, cesarean section), occurrence of eclampsia and postpartum hemorrhage (postpartum hemorrhage is defined as vaginal bleeding more than 500 ml within 24 h after delivery); recording of maternal lower limb muscle strength using the Bromage score at T_2_; recording the number of successful compressions and the total drug dosage administered by the analgesic pump; neonatal weight, Apgar score at 1, 5 and 10 min after birth were collected; the umbilical cord arterial blood gas analysis was also analyzed and recorded. The changes of TNF-α, IL-6, Cor in maternal venous blood were measured at T_0_, T_5_ and 24 h after delivery (T_7_).

### Statistical analysis

SPSS for Windows software package ver. 19.0 (SPSS, Inc., Chicago, IL, USA) was used for statistical analysis. Data was shown as mean ± SEM and analyzed using unpaired t-test. For categorical data like adverse events, Chi-square test was applied. In this study, *P* < 0.05 was accepted to be statistically significant.

## Results

### Patients’ characteristics

**Fig. 1:**
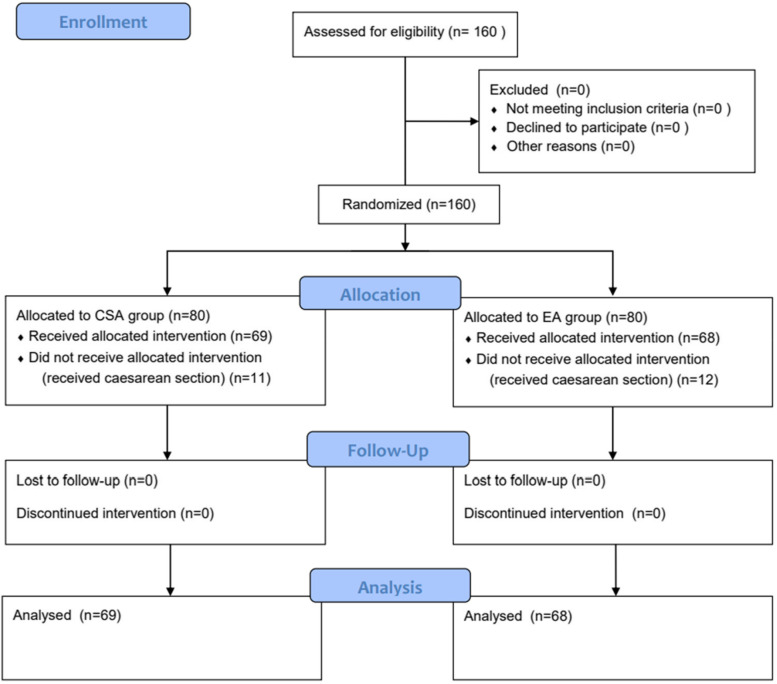
CONSORT 2010 guidelines

In this study, in total 80 parturients were included in CSA group, 8 parturients received caesarean section due to fetal distress, 3 parturients received caesarean section due to prolongation of the first stage of labor; 80 cases were included in EA group, 9 parturients received caesarean section due to fetal distress, 3 parturients received caesarean section due to prolongation of the first stage of labor. This clinical trial is in compliance with CONSORT 2010 guidelines (Fig. 1).

**Fig. 2:**
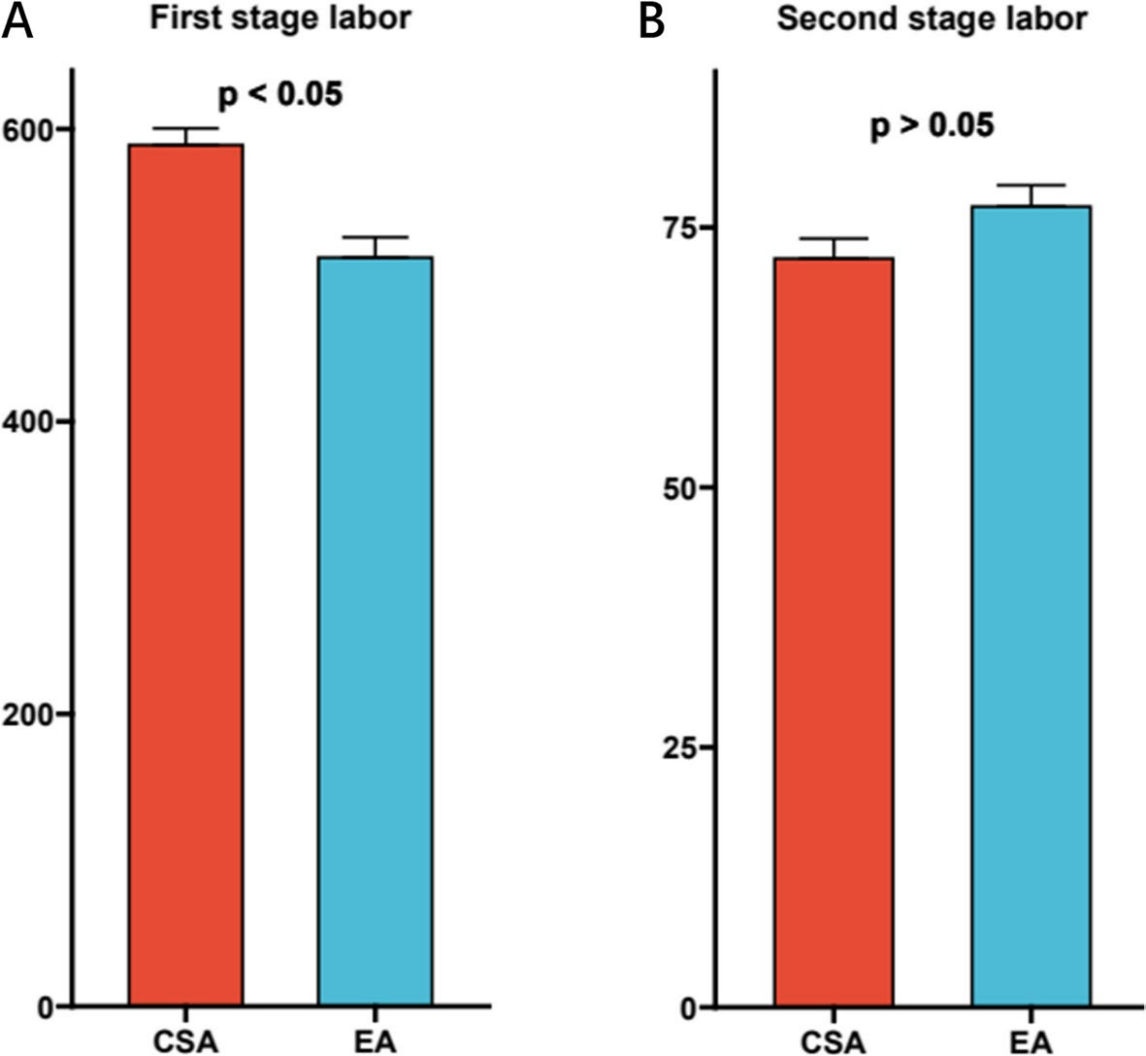
The duration for the first and second labor stage between CSA and EA group

We had compared parturients’ age, body weight, height, gestational weeks between CSA and EA group, but didn’t observe any difference, suggesting our study was unbiased (Table 1). We also compared the duration for the first and second labor stage between CSA and EA group respectively, and only noticed that the first labor stage was slightly prolonged in CSA group (Fig. 2a, b).

**Table 1:** Comparison of parturients’ age, body weight, height, gestational weeks between CSA and EA group

	Numbers	Age(years)	Height (cm)	Body weight (Kg)	Gestational weeks
CSA group	69	31.5 ± 3.8	161.2 ± 5.2	76.4 ± 7.0	37.6 ± 1.6
EA group	68	31.8 ± 4.0	160.3 ± 5.5	76.2 ± 7.0	37.7 ± 1.5

### Recording and comparison of hemodynamics between CSA and EA parturients

To comprehensively analyze the hemodynamics’ change during throughout the labor with different anesthesia methods, we recorded the mean arterial pressure (MAP), visual analogue scale (VAS), cardiac output (CO) and systematic vascular resistance (SVR) at different time points (T_1_-T_5_) for every parturient.

**Fig. 3:**
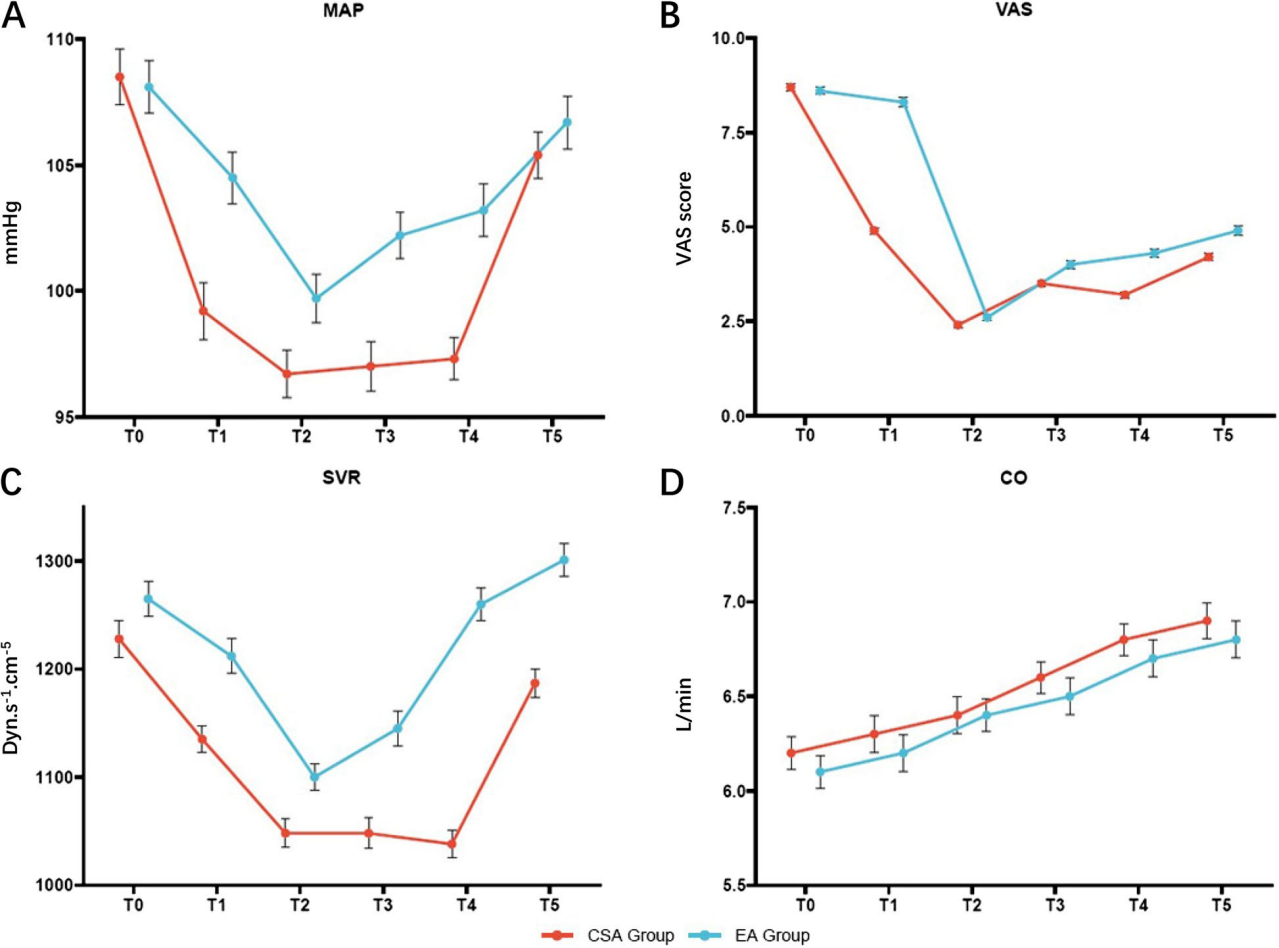
The MAP value, VAS score, SVR value, CO value between CSA and EA group

Our data showed that the MAP value was significantly decreased at T_1_、T_3_、T_4_ in CAS group as compared to the EA group (Fig. 3a), the VAS score and SVR value were both obviously lower in CSA group at T_1_, T_3_, T_4_ and T_5_ (Fig. 3b, c), in contrast, the CO value was higher in CSA group at T_3_ and T_4_ (Fig. 3d), suggesting different anesthesia methods might affect the hemodynamic of hypertensive parturients.

### Parturients with continuous spinal analgesia required more oxytocin but less antihypertensive drugs

**Fig. 4:**
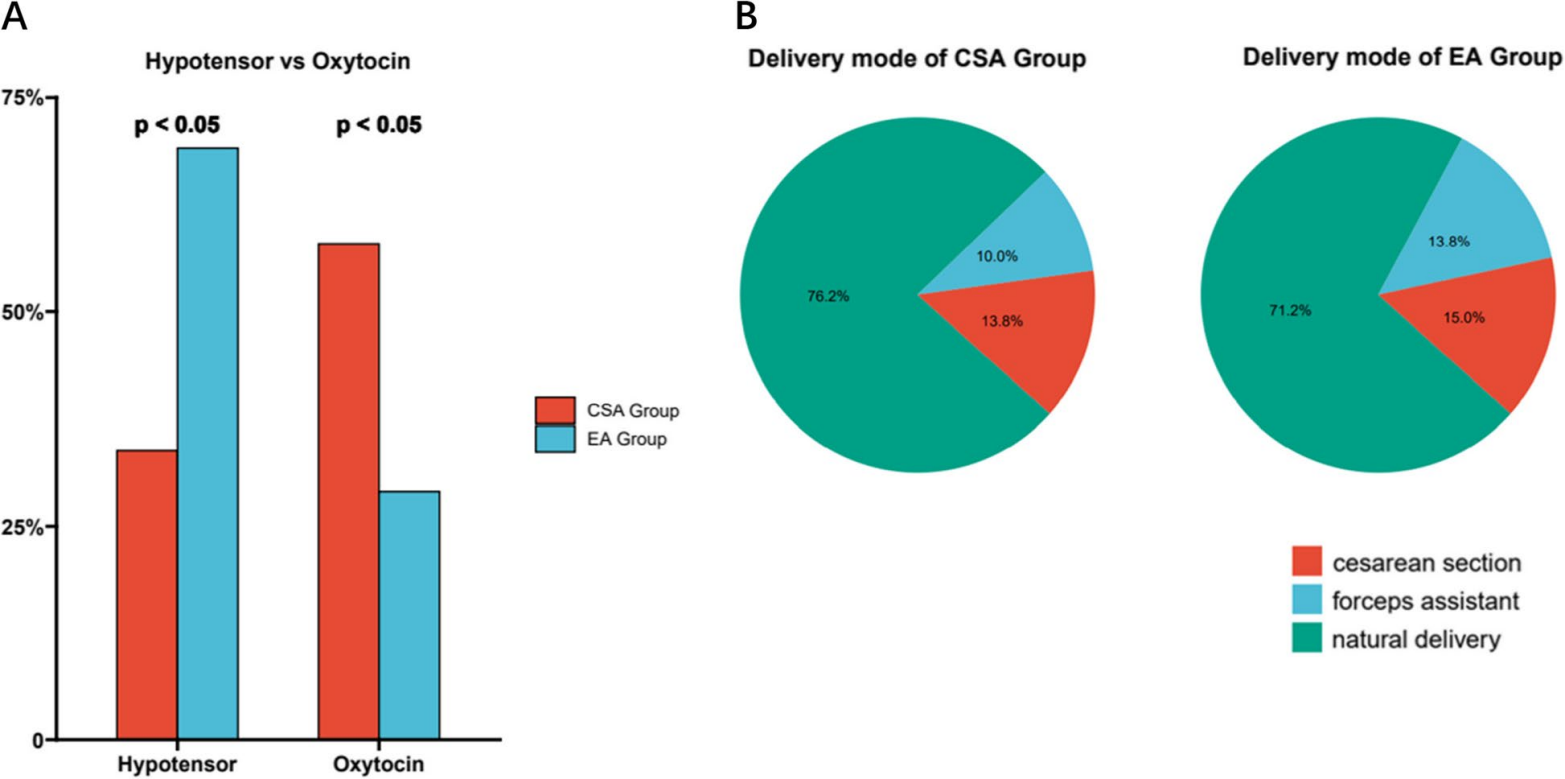
The usage frequency of oxytocin and antihypertensive drugs between CSA and EA group

We then compared the usage frequency of oxytocin and antihypertensive drugs during labor between CSA and EA group. Intriguingly, parturients in the CSA group demanded more oxytocin usage but significantly less antihypertensive drugs (Fig. 4a, b). Furthermore, both forceps assisted delivery and cesarean rate in the CSA group was slightly lower than that in the EA group, and most importantly, there was no case of eclampsia and postpartum hemorrhage occurred in either CSA or EA group. According to the follow-up on day 2 after delivery, no dural puncture (PDPH) occurred in the EA group, and only 1 patient in the CSA group had mild PDPH which resolved on day 55 after delivery. 4 patients in the CSA group had a modified Bromage score of 1 and the rest had a score of 0. Only 1 patient in the EA group had a modified Bromage score of 1 and the rest had a score of 0. Total drug dosage was lower in the CSA group compared with the EA group (*P* < 0.01) (Table 2).

**Table 2:** Comparison of the number of successful compressions and the total amount of medication used in the two groups of maternal analgesic pumps

	Numbers	Successful compressions	Total drug dosage (ml)
CSA group	69	4.79 ± 1.78	19.2 ± 8.41▲
EA group	68	5.98 ± 2.18	39.8 ± 11.8

Supplement: Compared with CSA group, ▲*P* < 0.01

### Continuous spinal analgesia had similar effect to the newborns as compared to continuous epidural analgesia

Our data showed that there was no significant difference in newborn body weight and Apgar scores at 1,5 and 10 min after birth between CSA and EA groups (Table 3). Furthermore, the neonatal umbilical cord arterial blood gas analysis indicated comparable effect of CSA and EA to the newborns. there was no significant difference in cord artery blood gas analysis between the two groups (Table 3).

**Table 3:** Comparison of newborns’ bodyweight, Apgar score and blood gas analysis of neonatal umbilical cord arterial blood between CSA and EA group

	Number	Newborns’ bodyweight	1 min Apgar	5 min Apgar	10 min Apgar
CSA	69	2920 ± 395	8.8 ± 0.7	9.2 ± 0.8	9.6 ± 0.5
EA	68	3105 ± 381	8.7 ± 0.7	9.2 ± 0.7	9.7 ± 0.5
		PH	PCO_2_(mmHg)	PO_2_(mmHg)	BE
CSA		7.33 ± 0.46	42.3 ± 5.4	58.5 ± 3.8	-3.8 ± 1.4
EA		7.31 ± 0.43	45.2 ± 5.3	58.0 ± 3.9	-3.6 ± 1.4

### Continuous spinal analgesia effectively reduces stress response and inflammatory factor release of parturients

By comparing the inflammatory response factors and stress response hormones in the serum of pregnant women in the labor process between CSA group and EA group (Table 4), we found that the level of TNF-α, IL-6, Cor in the CSA group at T_5_ was lower than that of EA group (*P* < 0.05), and the level of TNF-αin the CSA group at T_7_ was lower than that of EA group (*P* < 0.05).

**Table 4:** Comparison of serum stress response and inflammatory factor indexes between two groups of parturients ($\overline{\mathrm{x} }$ ± s)

	T0	T5	T7
TNF-α(ng/ml)			
CSA	2.22 ± 0.36	3.67 ± 0.72▲	3.12 ± 0.84▲
EA	2.14 ± 0.42	4.13 ± 0.57	3.34 ± 0.95
IL-6(ng/L)			
CSA	0.524 ± 0.614	0.639 ± 0.442▲	0.611 ± 0.586
EA	0.565 ± 0.577	0.678 ± 0.649	0.641 ± 0.687
Cor(nmol/L)			
CSA	551 ± 63	561 ± 50▲	519 ± 49
EA	526 ± 68	596 ± 55	531 ± 60

Supplement: Compared with EA group, ▲*P* < 0.05

## Discussion

The main pathological feature of hypertension in pregnancy is systemic arterial spasm [2, 13]. With the disease progression, the perfusion of various organs decreases, resulting ischemia and hypoxia damage to multiple tissues and often accompanied with cardiac insufficiency [14]. The parturient usually experiences a strong stress response due to tension, anxiety and pain stimulation during childbirth, which increases the release of catecholamines and lead to eclampsia or cardiovascular and cerebrovascular accidents [15]. The timely termination of pregnancy after systemic treatment is an effective method to prevent hypertension in pregnancy [16]. Currently, most clinicians choose using cesarean section to end childbirth directly, but this does not significantly improve the outcome of newborns, and selective cesarean section can induce neonatal respiratory distress syndrome, neonatal wet lung and other complications. Also, high incidence of hemorrhage and fatal amniotic fluid embolism were reported during cesarean section than vaginal delivery. Thus, it is important to explore better conditions to improve pregnancy outcomes for patients with gestational hypertension without indication of emergency cesarean section.

The implementation of labor analgesia can effectively maintain the stability of the circulation during the delivery process and reduce the occurrence of related complications [17]. However, maternal postural changes, effects of anesthesia, delivery of the fetus, and forceps usage can cause severe fluctuations in hemodynamics [18, 19]. Therefore, efficient monitoring methods and appropriate anesthesia management are essential for women with pregnancy-induced hypertension [20].

Continuous epidural anesthesia is a widely used method for labor analgesia, but the dose of drugs used in this way may lead to excessive maternal absorption of drugs into the bloodstream, causing respiratory depression in the newborns, and there is also incomplete analgesic block in some cases. Continuous subarachnoid anesthesia is delivering the anesthetic drugs through a micro catheter placed into the subarachnoid space directly. Compared with continuous epidural anesthesia, the multiple advantages of continuous subarachnoid anesthesia are small dosage, accurate effect, and stabilizing circulatory system, which is an ideal anesthesia strategy for pregnant women with hypertension as the dosage of ropivacaine is reduced, significant motor nerve block is rarely occurred [21].

The accurate hemodynamic monitoring system is also essential during the labor process [22]. The LiDCO-rapid is a non-invasive hemodynamic monitor that uses the principle of energy conversion in physics to convert the ejection volume power per stroke into electrical energy and record it, combining the patient’s blood pressure, heart rate, height, weight and other parameters to generate various hemodynamic indicators can be used in both mechanical and non-mechanical ventilation [8, 12]. The accuracy and reliability of the LiDCO-rapid monitoring data have been reproducibly confirmed in a lot of studies.

For those patients with continuous epidural analgesia, sufentanil is only used in the epidural space, the analgesic effect is not so good although the dosage is large, sufentanil can be absorbed into the blood through the venous plexus of the epidural space, increasing the risk of neonatal respiratory depression [23]; Ropivacaine not only blocks the sensory nerve, but also has certain blocking effect on the motor nerve, and even impairs the breath-holding force of the second stage of labor [24, 25]. In the current study, ropivacaine plus sufentanil were delivered either via continuous subarachnoid analgesia (CSA group) or continuous epidural analgesia (EA group), the MAP, VAS and SVR of both groups decreased, indicating that labor analgesia can alleviate the stress response of the parturients. Notably, the MAP, SVR, and VAS scores of the CSA group were lower than EA group 10 min after analgesia, indicating that continuous subarachnoid anesthesia had a faster onset than epidural anesthesia with better analgesia effect. Thirty minutes after analgesia, the MAP, VAS, and SVR of the EA group tended to be comparable with CSA, suggesting that epidural anesthesia could only achieve continuous subarachnoid analgesia 20–30 min after administration. However, the MAP, VAS and SVR of the EA group were significantly higher than CSA group when the uterus was opened and the fetus was delivered, indicating that deliver ropivacaine and sufentanil into the subarachnoid space could suppress the pain with longer duration, which significantly reduced the stress state of parturients and improved the perfusion of tissues and organs, thus meet the analgesic requirements of the first and second stages of labor. Importantly, obstetricians could adjust the application of antihypertensive drugs according to the parameters of the LiDCO-rapid monitor, which could effectively avoid hypotension and insufficient uterine perfusion. Our study therefore showed that the maternal hemodynamics in the CSA group was more stable throughout the labor process.

The first stage of labor in the CSA group was longer than EA group because ropivacaine partially blocked the visceral nerves, reduced the intensity of maternal uterine contractions, and reduced the frequency of uterine contractions. There was no significant difference between the duration of the second stage of labor in the CSA group and the EA group, suggesting that the combined application of ropivacaine and sufentanil for subarachnoid analgesia had less effect on the second stage of labor. In terms of adverse reactions, the application rate of oxytocin in the CSA group was higher, but the application rate of antihypertensive drugs was significantly lower than EA group, indicating that the subarachnoid application of ropivacaine combined with sufentanil had a stronger analgesic effect. Compared with traditional epidural labor analgesia, continuous subarachnoid analgesia had no significant effect in the final delivery method. The newborns in the two groups had similar Apgar scores at 1, 5, and 10 min, and no significant respiratory depression occurred. The results of umbilical arterial blood gas analysis of newborns in both groups were all within the normal range, and there was no obvious acidosis.

Previous studies have shown that both hypertension in pregnancy and normal delivery induce protective mechanisms such as stress and inflammatory responses, but overexpression of stress and inflammatory responses can impair maternal recovery. Cor is a stress-responsive hormone that is released in large amounts during trauma. In this study, the level of Cor in the CSA group at T_5_ was lower than the EA group, suggesting that continuous subarachnoid anesthesia can better suppress the stress response effectively. TNF-α and IL-6 are pro-inflammatory response factors, and two indicators in the body trauma can indicate the degree of inflammatory response of the body. Hypertension in pregnancy and childbirth all lead to increased maternal TNF-α and IL-6 levels. In this study, TNF-α and IL-6 levels in the serum were lower in the CSA group than the EA group at T_5_, and TNF-α in the serum was lower in the CSA group than the EA group at T_7_, suggesting that continuous subarachnoid anesthesia is more effective in suppressing the inflammatory response.

Taken together, for pregnant women with pregnancy induced hypertension, continuous subarachnoid analgesia can improve the oxygen supply to the fetus with better analgesia effect and stable circulatory system without significant effect to the final delivery method and newborns, but it can effectively reduce stress response and inflammatory factor release of parturients therefore serves as an ideal anesthesia method for parturients with hypertension.

One limitation of this study is that our study was not blinded, and the sample size included in our study is too small, and there may be some deviations in the prospective analysis; Another limitation was the anesthesia method in the study is not very commonly used clinically.

## Data Availability

The datasets used and analyzed during the current study are available from the corresponding author upon reasonable request.

## Abbreviations

CSA: Continuous spinal analgesia group
EA: Continuous epidural analgesia group
